# Screening tools to address social determinants of health in the United States: A systematic review

**DOI:** 10.1017/cts.2024.506

**Published:** 2024-04-05

**Authors:** Mahdi Neshan, Vennila Padmanaban, Diamantis I. Tsilimigras, Samilia Obeng-Gyasi, Naleef Fareed, Timothy M. Pawlik

**Affiliations:** 1 Department of General Surgery, Shahid Sadoughi University of Medical Sciences and Health Services, Yazd, Iran; 2 Department of Surgery, The Ohio State University Wexner Medical Center and James Comprehensive Cancer Center, Columbus, OH, USA; 3 Department of Biomedical Informatics, College of Medicine, The Ohio State University, Columbus, OH, USA

**Keywords:** Health disparities, needs assessment, screening, social determinants of health, SDoH

## Abstract

The Centers for Medicare & Medicaid Services have mandated that hospitals implement measures to screen social determinants of health (SDoH). We sought to report on available SDoH screening tools. PubMed, Scopus, Web of Science, as well as the grey literature were searched (1980 to November 2023). The included studies were US-based, written in English, and examined a screening tool to assess SDoH. Thirty studies were included in the analytic cohort. The number of questions in any given SDoH assessment tool varied considerably and ranged from 5 to 50 (mean: 16.6). A total of 19 SDoH domains were examined. Housing (*n* = 23, 92%) and safety/violence (*n* = 21, 84%) were the domains assessed most frequently. Food/nutrition (*n* = 17, 68%), income/financial (*n* = 16, 64%), transportation (*n* = 15, 60%), family/social support (*n* = 14, 56%), utilities (*n* = 13, 52%), and education/literacy (*n* = 13, 52%) were also commonly included domains in most screening tools. Eighteen studies proposed specific interventions to address SDoH. SDoH screening tools are critical to identify various social needs and vulnerabilities to help develop interventions to address patient needs. Moreover, there is marked heterogeneity of SDoH screening tools, as well as the significant variability in the SDoH domains assessed by currently available screening tools.

## Introduction

Social determinants of health (SDoH) are conditions in which individuals are born, reside, engage in employment, acquire knowledge, practice religion, enjoy recreational activities, and grow old [1]. Taken together, SDoH are a well-established classification of essential non-medical factors that directly or indirectly impact health outcomes [2,3]. These factors may impact access to health care and may be related to individual behaviors as well as disease biology with important implications to an individual’s health [2,3]. In addition, the COVID-19 pandemic highlighted how patients in vulnerable socioeconomic contexts were at heightened risk of disease transmissibility, hospitalization, and mortality [4]. In response to the exacerbation of longstanding health disparities during the pandemic, there has been an increased interest in methods to identify and define SDoH [4,5]. By accessing data on SDoH, there is the potential to implement policies and target interventions to address barriers to health and healthcare delivery [6]. Importantly, resolving unmet social needs that underpin SDoH represents an opportunity to meaningfully improve population health, quality of life, and life expectancy, as well as patient outcomes [7].

Personal and systemic factors compromise a wide range of social determinants of health that drive health outcomes [8–12]. The World Health Organization (WHO) classifies SDoH into five broad domains: economic stability, education, social and community context, health care access and quality, and neighborhood and built environment [13]. In addition to these broad domains, additional dimensions include – but are not limited to – race and ethnicity, housing, food security, transportation, violence and safety, employment, health behaviors (i.e., substance use, physical activity, and dietary choices), mental health, disabilities, religion, immigration status, legal concerns, gender, and sexual orientation.[14,15] For instance, substandard housing has been associated with a higher prevalence of respiratory, hematologic, and neurologic illness, as well as childhood lead poisoning [16].

The COVID-19 pandemic exacerbated healthcare disparities [17,18], drawing attention to the need to develop federal and community-based policies to improve health equity. The recently issued United States Domestic Policy Counsel Playbook outlined recommendations for federal agencies to improve policies around SDoH with an emphasis on identifying social metrics relevant to health outcomes [17]. The Playbook served as a call to stakeholders and agencies to develop actionable programmatic changes to quantify and improve SDoH metrics. Proposed reforms are intended to occur at the federal and local levels to support community organizations to institute patient-level screening. These broad changes also seek to achieve a secondary goal: easing the substantial economic burden of health expenditures that occur due to pervasive health inequity [19,20]. Concurrent with these initiatives, the Centers for Medicare & Medicaid Services (CMS) have mandated that hospitals implement two new measures in 2024 to screen patients for SDoH: SDoH-1 or Screening for Social Drivers of Health and SDoH-2 or Screen Positive Rate for Social Drivers of Health [21]. While mandating reporting of SDoH measures, CMS does not offer uniform data-capturing methods/approaches, instead giving hospitals flexibility/discretion in how SDoH characteristics are recorded.

Screening tools intended to capture data on SDoH can vary significantly with vastly different domains, which may complicate how data are collected and used to develop community interventions to address health inequities [22,23]. Therefore, the objective of the current study was to report on available SDoH screening tools in a systematic manner aimed at addressing disparities identified using these tools.

## Methods

### Search methods

The study adhered to the Preferred Reporting Items for Systematic Reviews and Meta-Analysis (PRISMA) guidelines. This systematic review protocol was registered with PROSPERO, an internationally recognized database for prospectively registered systematic reviews in the fields of health and social care [24]. A comprehensive search of the PubMed, Scopus, and Web of Science databases from 1980 to November 2023 was performed using predetermined keywords. The search included a mix of subject headings and keywords that related to different social determinants screening tools, as well as specific proposed SDoH addressing interventions (Table 1). In addition to searching PubMed, Scopus, and Web of Science databases, a search of “grey literature” sources was also performed based on references of relevant studies, as well as an international clinical trials registry platform to identify parallel and ongoing research. Inclusion criteria included: (a) written in the English language, (b) conducted in the United States, and (c) established a screening tool to identify or address SDoH. Reviews and reports with no publicly accessible survey tool were excluded. Studies that fulfilled inclusion criteria reported data from 2007 to 2023, and each study provided an SDoH screening tool or an SDoH intervention. All reports initially identified from the database search were entered into ENDNOTE software for analysis.

**Table 1.** Search strategy and keywords used for literature screening

Database	Number
PubMed/Medline: ((“Recommended screening tool”[Title] OR “recommended screening tool”[Title] OR Screen [Title] OR screening [Title] OR Address [Title] OR Addressing [Title] OR tool [Title] OR toolkit [Title] OR intervention [Title] OR interventional [Title]) OR (“Address” [Publication Type])) AND ((“Social Determinants of Health”[Mesh]) OR (“Social determinants of health”[Title] OR “social determinants”[Title] OR SDoH [Title] OR “health-related social conditions”[Title]))	806
Scopus: (TITLE (“Recommended screening tool” OR “recommended screening tool” OR screen OR screening OR address OR addressing OR tool OR toolkit OR intervention OR interventional) AND TITLE (“Social determinants of health” OR “social determinants” OR SDoH OR “health-related social conditions”))	639
ISI Web of Science: “Recommended screening tool” OR “recommended screening tool” OR Screen OR screening OR Address OR Addressing OR tool OR toolkit OR intervention OR interventional (Title) AND “Social determinants of health” OR “social determinants” OR SDoH OR “health-related social conditions” (Title)	676
https://trialsearch.who.int/	3
Included investigations references	9

## Results

### Study characteristics

**Figure 1.**
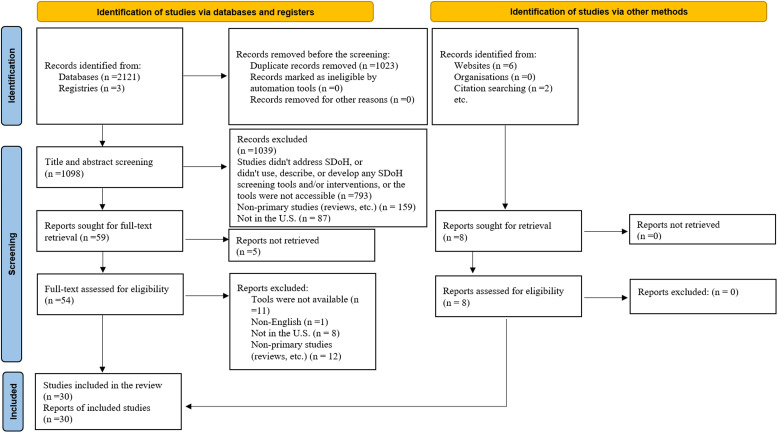
Preferred reporting items for systematic reviews and meta-analysis (PRISMA) demonstrating selection of studies included in the analytic cohort.

The initial search identified 2,121 studies. After eliminating duplicate entries, a total of 1,098 studies underwent primary screening. Following title and abstract screening, studies that did not address SDoH, or did not propose any publicly accessible SDoH screening tools and/or interventions (*n* = 793), were excluded. In addition, non-primary studies (reviews, etc.) (*n* = 159) and studies that were not conducted in the USA (*n* = 87) were excluded. A total of 59 studies were sought for full-text retrieval. Following a secondary review of these 59 full texts, 22 studies were deemed eligible for inclusion [13,25–45]. Following a manual search of the literature, as well as after snowballing the citations of included studies, 8 additional articles were incorporated into the review [46–53]. As such, a total of 30 papers were included in the analytic cohort (Fig. 1).

### Screening tools characteristics

Table 2 describes the SDoH screening tool characteristics of the 25 unique screening tools that were identified. Six screening tools were administered to pediatric patients [27,29–31,37,47]. One was designed to assess pregnant patients [34], and the remaining tools (*n* = 18) were utilized for general screening purposes in clinical settings, such as hospitals or clinical offices [13,25,26,28,32,33,35,36,38–46,48–53] Six tools were administered by healthcare professionals [13,25,30,32,46,49]; while 12 tools were completed by patients (or parents) either electronically or on paper [13,28,29,34,36,37,44,45,47,48,50,52]; six tools were administered verbally or were self-administered at the patient’s request [26,27,31,33,35,53]; The number of questions in any given SDoH assessment tool varied considerably and ranged from 5 in Health Leads (2018) and the North Carolina toolkit [36], as well as the Core 5 social risk tool [28] to 50 in the health system’s EPIC electronic health records screening tool [33]; overall, the mean number of questions in any given SDoH screening tool assessment was 16.6 (Table 2).

**Table 2.** SDoH screening tool characteristics of the 25 unique screening tools that were identified

Number	Tool/ Study	Setting (type of application)	Who/how administered/number of questions
1	PRAPARE* [25]	Clinical settings	Physician or nurse practitioner, computer-based/ 12 + demographic
2	AHC HRSN * [27]	General Pediatrics Clinic	Self-administered or Verbal with outreach coordinators/10
3	de Ramirez et al [13]	Primary care offices	In-person questionnaire/22
4	NLP-based method* [13]	Referral hospital system	Passive identification of SDoH through NLP /Non
5	WE CARE [26]	Urology Clinic	Self-administered or verbal /10
6	Health Leads (2018) and the North Carolina [36]	Community-based MHC*	Paper patient intake/5
7	Macias-Konstantopoulos et al [35]	Single academic medical center	Verbal or electronic /16
8	SIPT* ^34^	Pregnant patients	Paper patient intake /32
9	Health system’s EPIC EHRs* (version 2019) [33]	Outpatient setting at a cancer center	Electronic (REDCap) + Verbal /50
10	Gupta et al [32]	Patients (+ 18y) engaged in community health, inpatient, or ambulatory	Verbal /13
11	Friedman et al [30]	Pediatric Resident Clinic	Asked by the physician during the visit /10
12	Core 5 Social Risk Screening [28]	Clinical practice	Self-administered /5
13	Sokol et al [37]	Outpatient pediatric patients	Self-administered /12
14	I screen [31]	Pediatric emergency department	Self-administered or face-to-face with research assistant/23
15	AHC HRSN (modified with exploring perceived acceptability of screening) [29]	Pediatric patients of primary care clinics and emergency departments	Self-administered using a tablet device/32
16	Well Rx [50]	Clinical setting	Self-administered/11
17	Health begins [49]	Clinic settings	Student, health care staff, or provider; Paper/ 29
18	Health leads [48]	Clinical settings	Self-administered /9
19	Help Steps [47]	Children’s Hospital + Public Health Commission	Web-based/ 12
20	The Every ONE Project [46]	Clinical setting	Health care providers/ 14
21	North Carolina Toolkit [51]	Clinical setting	Not mentioned/11
22	IHELP* [45]	Clinical setting	Self-administered /13
23	MASQ* [44]	Clinical setting	Self-administered /10
24	Bright Future [52]	Clinical setting	Self-administered /45
25	Montefiore’s Survey [53]	Clinical setting	Self-administered or verbal /10

SDoH = social determinants of health; PRAPARE = Protocol for Responding to and Assessing Patient Assets, Risks, and Experiences; AHC = Accountable Health Communities; HRSN = Health-Related Social Needs; NLP = natural language processing; MHC = Mobile Health Clinic; WE CARE = Welcome, Engage, Communicate, Ask, Reassure, Exit; SIPT = Social Determinants of Health in Pregnancy Tool; EHRs = electronic health records; IHELP = Income, Housing, Education, Legal Status, Literacy, Personal Safety; MASQ = Medical-legal Advocacy Screening Questionnaire.

### Tools comprehensiveness

**Figure 2.**
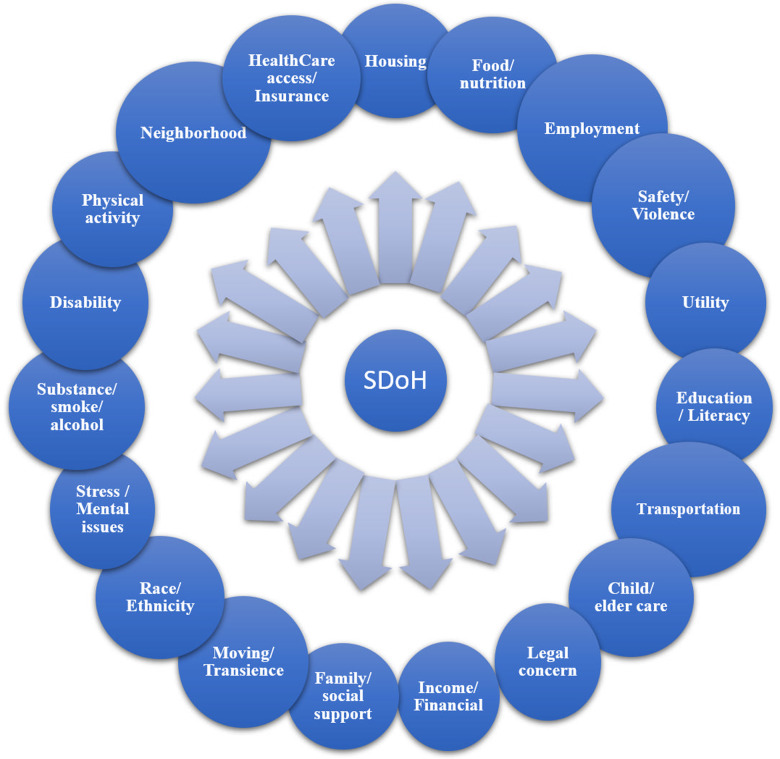
Various SDoH domains that may impact patient health. SDoH = social determinants of health.

**Figure 3.**
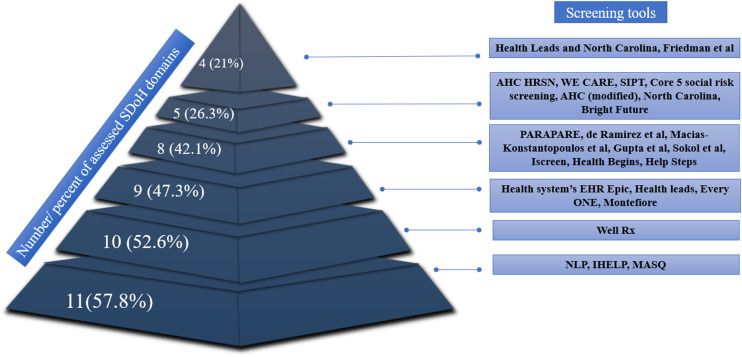
Relative number of SDoH domains assessed in the various screening tools. SDoH = social determinants of health; AHC = Accountable Health Communities; HRSN = Health-Related Social Needs; WE CARE = Welcome, Engage, Communicate, Ask, Reassure, Exit; SIPT = Social Determinants of Health in Pregnancy Tool; PRAPARE = Protocol for Responding to and Assessing Patient Assets, Risks, and Experiences; EHRs = electronic health records; NLP = natural language processing; IHELP = Income, Housing, Education, Legal Status, Literacy, Personal Safety; MASQ = Medical-legal Advocacy Screening Questionnaire.

A total of 19 distinct SDoH domains were examined in the various screening tools (Fig. 2). Various screening tools evaluated different domains, ranging from four domains (21%) in Health Leads and the North Carolina [36], and Friedman et al. screening tools [30], to 11 (57.8%) within the natural language processing (NLP) [13], Income, Housing, Education, Legal status, Literacy, Personal safety (IHELP) [45], and Medical-legal advocacy screening questionnaire (MASQ) [44] tools (Fig. 3). The Well Rx tool [50] evaluated 10 SDoH domains (52.6%), while the EPIC EHR [33], Health leads [48], EveryONE project [46], and Montefiore [53] tools evaluated 9 domains (47.3%). Eight SDoH domains (42.1%) were evaluated in Protocol for Responding to and Assessing Patient Assets, Risks, and Experiences (PARAPARE) [25], as well as the tools proposed by de Ramirez *et al*., [13] Macias-Konstantopoulos *et al*., [35] Gupta *et al*., [32] Sokol *et al*. [37] The Iscreen [31]. Health Begins [49], Help Steps surveys [47]. Tools such as Accountable Health Communities Health-Related Social Needs (AHC HRSN) [27], Welcome, Engage, Communicate, Ask, Reassure, Exit (WE CARE) [26], Social Determinants of Health in Pregnancy Tool (SIPT) [34], Core 5 social risk screening [28], Accountable Health Communities (modified) [29], North Carolina [51], and Bright Future [52] examined 5 domains of SDoH (26.3%). Four screening tools (16%) evaluated at least 10 (52.6%) different SDoH domains (Well Rx [50], NLP [13], IHELP [45], MASQ [44]), while the remaining screening tools (*n* = 21, 84%) evaluated fewer SDoH domains (Table 3).

**Table 3.** Domains assessed by each screening tool

Domains Tools (studies)	Housing	Food/ Nutrition	Employment	Safety/Violence	Utility	Education/ Literacy	Transportation	Child/ elder care	Legal concern	Income/Financial	Family/social support	Moving/Transience	Race/ Ethnicity	Stress /Mental issues	Substance/ smoke/ alcohol	Disability	Physical activity	Neighborhood	HealthCare access/ Insurance
PRAPARE* [25]	✓		✓	✓		✓			✓	✓	✓		✓						
AHC HRSN* [27]	✓	✓		✓	✓		✓												
de Ramirez et al [13]	✓	✓		✓		✓	✓			✓	✓			✓					
NLP-based method* [13]	✓	✓	✓	✓			✓			✓	✓			✓	✓	✓			✓
WE CARE* [26]	✓	✓		✓	✓		✓												
Health Leads and the North Carolina [36]	✓	✓			✓		✓												
Macias-Konstantopoulos et al [35]	✓	✓	✓		✓	✓	✓	✓		✓									
SIPT* [34]				✓						✓	✓			✓	✓				
Health system’s EPIC EHRs* [33]	✓	✓		✓			✓			✓	✓			✓	✓		✓		
Gupta et al [32]	✓	✓		✓	✓	✓	✓			✓	✓								
Friedman et al [30]	✓			✓							✓				✓				
Core 5 Social Risk Screening [28]	✓	✓			✓		✓				✓								
Sokol et al [37]	✓	✓	✓		✓	✓	✓	✓		✓									
I screen [31]	✓		✓	✓		✓			✓	✓		✓	✓						
AHC HRSN* (modified) [29]	✓	✓		✓	✓		✓												
Well Rx [50]	✓	✓	✓	✓	✓	✓	✓	✓		✓					✓				
Health begins [49]	✓		✓	✓		✓			✓	✓	✓	✓							
Health leads [48]	✓		✓	✓		✓			✓	✓	✓	✓	✓						
Help Steps [47]	✓		✓	✓		✓			✓	✓	✓		✓						
The Every ONE [46]	✓	✓	✓	✓	✓	✓	✓	✓		✓									
North Carolina [51]	✓	✓		✓	✓		✓												
IHELP* [45]	✓	✓		✓	✓	✓		✓		✓	✓				✓			✓	✓
MASQ* [44]	✓	✓		✓		✓		✓	✓	✓				✓	✓			✓	✓
Bright Future [52]				✓							✓			✓	✓			✓	
Montefiore [53]	✓	✓		✓	✓		✓	✓	✓		✓								✓

PRAPARE = Protocol for Responding to and Assessing Patient Assets, Risks, and Experiences; AHC = Accountable Health Communities; HRSN = Health-Related Social Needs; NLP = Natural Language Processing; MHC = Mobile Health Clinic; WE CARE = Welcome, Engage, Communicate, Ask, Reassure, Exit; SIPT = Social Determinants of Health in Pregnancy Tool; EHRs = Electronic Health Records; IHELP = Income, Housing, Education, Legal status, Literacy, Personal Safety; MASQ = Medical-legal Advocacy Screening Questionnaire.

### SDoH domains

**Figure 4.**
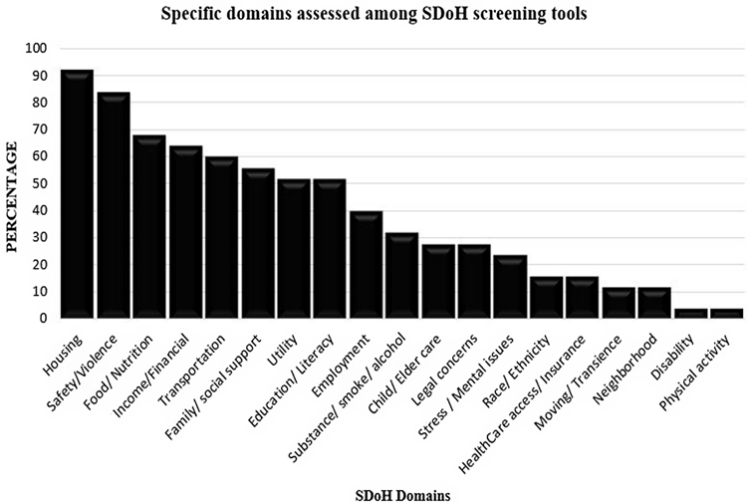
Specific SDoH domain themes that were assessed among the different SDoH screening tools. SDoH = social determinants of health.

While no individual SDoH domain was assessed in every screening tool, housing (*n* = 23, 92%) and safety/violence (*n* = 21, 84%) were the domains assessed most frequently examined (Fig. 4). SDoH domains involving food/nutrition (*n* = 17, 68%), income/financial (*n* = 16, 64%), transportation (*n* = 15, 60%), family/social support (*n* = 14, 56%), utilities (*n* = 13, 52%), and education/literacy (*n* = 13, 52%) were also commonly included in most SDoH screening tools. Other SDoH domains that were commonly assessed in the various screening tools included employment (*n* = 10, 40%), substance/smoke/alcohol use (*n* = 8, 32%), stress/mental issues (*n* = 6, 24%), child/elder care (*n* = 7, 28%), and legal concerns (*n* = 7, 28%). In contrast, race/ethnicity (*n* = 4, 16%), healthcare access/insurance (*n* = 4, 16%), moving/transience (*n* = 3, 12%), neighborhood (*n* = 3, 12%), disability (*n* = 1, 4.0%), and physical activity (*n* = 1, 4.0%) were the least commonly assessed domains among the different SDoH screening tools.

### SDoH-based interventions

Of note, 18 studies not only screened SDoH but also proposed specific interventions aimed at addressing SDoH (Supplemental Table 1) [13,26–28,30,32–34,36–43,47,50]. Twelve screening tools identified patient preferences toward receiving supplementary assistance relative to the SDoH identified. If the response was affirmative, referral to relevant social workers was made based on the positive domain that had been identified on screening [13,26–28,30,32–34,36,37,39,50]. Interestingly, three separate studies proposed interventions grounded in sports [38], sleep health [40], and developing a national agenda aimed at homelessness and homeless individuals to address SDoH [43]. Fleegler *et al*. had patients utilize a web-based application entitled Help Steps to not only self-identify social needs but also identify community-based support for those needs identified [47]. In a different study, Hassan et al. implemented a web-based tool for patients to assess SDoH domains, offering feedback and assistance in choosing appropriate agencies, and follow-up using phone calls [41]. In another study, Hatef *et al*.[42] developed an electronic health record (EHR)-derived community health record that aggregated data at both the hospital and neighborhood level as a means to capture local community health data at the population level, identify SDoH needs, and then link community-based resources to address patient needs.

## Discussion

SDoH represents a broad array of domains that can impact a patient’s lived experiences, including their overall well-being and health. Growing evidence has demonstrated that addressing unmet health-related social needs such as hunger, exposure to violence, homelessness, and transportation can help improve well-being [54]. While health providers routinely use clinical assessment algorithms, tools to screen SDoH have not been as widely adopted or implemented [28]. Collection of such data may inform patient treatment plans and referrals to community services [55]. When patients screen positive for particular social risks and social needs, targeted interventions may help address disparities and improve health equity. As such, CMS has mandated that hospitals screen patients for SDoH [21]. The means and methods to capture these data have not been well defined, however, with no single screening tool being universally adopted or available. The current study was important as we performed a systematic review of various screening tools published in the literature to identify and target SDoH in the clinical setting. Of note, the various screening tools were heterogeneous in their use, application, scope of inquiry, and targeted domains of SDoH. Many of the screening tools included a different number of SDoH domains, as well as variable domain types. Specifically, the median number of domains evaluated in SDoH screening tools was 8.0 (interquartile range, 9.0-5.0) with housing and safety/violence being the domains assessed most frequently (Fig. 4). Food/nutrition, income/financial, transportation, family/social support, utilities, and education/literacy were also commonly included in many SDoH screening tools. While less frequent, some reports utilized the SDoH identified in the screening tools to inform some type of intervention. For instance, sports-based interventions were proposed to improve personal physical and psychosocial health [38], while other studies proposed web-based applications and/or linking the EMR to community databases to identify community-based support for those needs identified on screening [41,42,47].

Among the tools with explicitly defined criteria, the NLP [13], IHELP [45], and MASQ [44] screening tools were the most comprehensive in their approach as these tools included the highest number of SDoH domains. The NLP algorithm system utilized the existing electronic medical record and identified keywords or phrases that suggested housing or financial needs (i.e., lack of permanent address); the NLP model performed with high accuracy. NLP combines computational linguistics with machine and deep learning models [56]. In turn, large amounts of EMR text data can be processed to understand its meaning and identify different themes including the risk of adverse SDoH. Adverse social SDoH may include social risks associated with poor health (e.g., food insecurity), and individual preferences and priorities regarding seeking assistance to address the social needs (e.g., seeking food assistance) [57]. An NLP approach is limited, however, in that it can only assess textual data that had been recorded in the EMR by healthcare providers. In contrast, SDoH screening questionnaires provide an opportunity to query patients specifically about different SDoH domains. The IHELP questionnaire focused on pediatric patients and queried SDoH domains such as income, housing/utilities, education, legal status/immigration, literacy, and personal safety [45]. In turn, data collected from this questionnaire may elicit specific environmental, legal, and psychosocial risk factors that can be utilized by social workers to address the needs of individual patients. For example, the use of the MASQ screening tool was able to identify families of pediatric patients who required assistance with legal services and help facilitate a referral [44]. Therefore, the use of screening tools can pinpoint the different SDoH domains needed by patients to allocate limited resources to serve that specific need.

Several tools such as the Health Leads and the North Carolina survey [36], as well as the screening tool proposed by Friedman *et al*., [30] focused on four domains including housing, safety/violence, family/social support, and substance/smoke/alcohol misuse. Other tools concentrated on screening for economic stability, education access and quality, health care access and quality, neighborhood and built environment, as well as social and community context [45,53]. Of note, IHELP was the only screening tool that addressed all five main SDoH domains identified by WHO [13,45]. Housing and safety/violence were the most frequently assessed domains among the screening tools. These SDoH themes highlight how housing insecurity plays a significant role in health status as overcrowding, frequent relocation, and housing expenses can negatively impact health [58,59]. In turn, helping patients secure housing can improve health through multiple mechanisms, including increasing patient safety [43]. Exposure to unsafe environments can have long-term health consequences, including amplifying chronic diseases and mental illnesses [60]. In addition, themes of food/nutrition, income/financial concerns, as well as transportation and education/literacy were other frequently evaluated domains across various assessment tools (Table 3). Interestingly, although repeatedly associated with increased risk of social vulnerability and adverse SDoH, race/ethnicity was often not included in screening tools – perhaps because these data are required already as part of the “meaningful use” of electronic health records [61].

Beyond proposing and implementing screening tools, several authors proposed interventions to address adverse SDoH. Overall, a total of 18 interventions in addition to primary SDoH screening were identified (Supplemental Table 1). Most interventions consisted of referring individuals to social health workers, who were selected based on the specific SDoH identified through the screening process. For example, Fleegler et al. used the SDoH screening tool to identify specific patient needs and then delivered assistance using a web-based application, which recommended specific community-based agencies [47]. In a similar manner, Hassan et al. proposed a different web-based tool that provided patient feedback and assistance in choosing appropriate agencies based on the SDoH screening tool as well as performing follow-up using telephone calls [41]. Utilization of web-based tools may serve to connect patients to resources based on needs identified through SDoH screening. Web-based tools may need to be supplemented, however, with patient navigators, lay community health care workers, as well public health workers who can serve as a bridge between communities, health care systems, and state health departments.

One of the main strengths of this review is that no other study has performed a thorough evaluation and comparison of available screening tools to address SDoH in the United States to date. Nevertheless, due to the heterogeneity of the tools and the diverse target populations evaluated by each individual screening tool, future efforts should aim at defining best practices in collecting SDoH, as well as identifying standardized means to report SDoH in a timely manner. In addition, despite the available screening tools, future efforts should aim at not only reporting but also addressing social needs and mitigate disparities in access to high-quality care.

In conclusion, CMS has mandated evaluation of SDoH to identify medical and social barriers that impede the health and well-being of patients [21]. SDoH and associated health disparities are important drivers of healthcare access and outcomes [62]. SDoH screening tools are critical to identify various social needs and vulnerabilities so that patients can be connected to effective interventions to address their needs [63]. The current systematic review demonstrated the heterogeneity of currently available SDoH screening tools, as well as the variability in the SDoH domains assessed. The use of technology via web-based screening platforms and the electronic medical records is critical to capture patient SDoH, as well as potentially link individuals with community resources. Patient navigators and public health community workers also play an important role in connecting patients with resources.

## Supporting information

Neshan et al. supplementary material Neshan et al. supplementary material

## Supplementary material

The supplementary material for this article can be found at https://doi.org/10.1017/cts.2024.506.
