# Supplementary material for: Screening tools to address social determinants of health in the United States: A systematic review
Source: J Clin Transl Sci. 2024 Apr 5;8(1):e60. doi: 10.1017/cts.2024.506 (PMC11036426; doi:10.1017/cts.2024.506)
Supplement: Neshan et al. supplementary material [file S2059866124005065sup001.docx]

**Supplemental Table 1.** Examples of possible interventions used to address SDoH.

| **Study** | **Intervention** |
| --- | --- |
| Elsborg et al ^1^ | Engage people in sports-based recreation activities. These activities should be free of charge, based on no or few facilities, locally available, and be tailored to the target groups. |
| Gurewich et al ^2^ | Identify unmet social needs and refer to social services to address unmet social needs |
| Hale et al ^3^ | considering improving sleep health a necessary step toward achieving health equity as sleep is a key indicator of overall health |
| Hassan et al ^4^ | Researchers developed a web-based tool for patients to assess health-related social domains, offering feedback and assistance in choosing appropriate agencies. Telephone follow-ups were conducted after 1-2 months to ensure patients had addressed their issues. |
| Hatef et al ^5^ | Proposing to develop an EHR*-derived CHR* which has the potential to significantly enhance the scope, accuracy, and timeliness of data accessible for designing interventions aimed at addressing SDoH at both individual and local or even national levels. |
| Henwood et al ^6^ | Developing a national agenda on homelessness and health disparities |
| Yaun et al ^7^ | Identify the most pressing need and work to meet that need directly through clinic resources, Methodist Le Bonheur Community Outreach services, or a network of community partners. |
| de Ramirez et al ^8^ | Patients were asked whether they would be willing to accept assistance if they screened positive for 1 or more SDoH categories, and results from the SDoH screening were incorporated into the patient's EHR |
| Roebuck et al ^9^ | A patient with positive SDoH results receives a community resource guide and discusses options. If needed, a nurse or medical assistant can initiate a referral for the patient to be connected with a patient navigator. |
| Mullen et al ^10^ | If the user has indicated "YES" for any of the options in the survey, it would be asked whether they wish to receive support for any of these identified needs to address them and refer patients to appropriate community services |
| Harriett et al ^11^ | Patients who screened positive for any section of SIPT were contacted to assess the linkage of services depending on each positive domain (mostly includes, counseling, referral, and handouts offer) |
| Hao et al ^12^ | The social work team addresses SDOH needs upon referral |
| Gupta et al ^13^ | Patients receive referrals based on the positive domain |
| Friedman et al ^14^ | Specific interventions and referrals for each positive domain |
| Bradywood et al ^15^ | Refer to social work by a registered nurse, social work connects with patients for individualized resource support and additional needs |
| Page-Reeves et al ^16^ | Refer to community health workers |
| Fleegler et al ^17^ | In the web-based context, patients could explore and choose the domain that needs to be addressed |
| Sokol et al ^18^ | Patients were asked whether they needed any assistance/ referral program for help on any of their responses/ domains |

*Abbreviations: EHR (Electronic Health Records), CHR (Community health records).

**References**

**1.** Elsborg P, Nielsen G, Klinker CD, Melby PS, Christensen JH, Bentsen P. Sports-based recreation as a means to address social inequity in health: why, when, where, who, what, and how. *BMC Public Health.* Aug 9 2019;19(1):1084.

**2.** Gurewich D, Garg A, Kressin NR. Addressing Social Determinants of Health Within Healthcare Delivery Systems: a Framework to Ground and Inform Health Outcomes. *J Gen Intern Med.* May 2020;35(5):1571-1575.

**3.** Hale L, Troxel W, Buysse DJ. Sleep Health: An Opportunity for Public Health to Address Health Equity. *Annu Rev Public Health.* Apr 2 2020;41:81-99.

**4.** Hassan A, Scherer EA, Pikcilingis A, et al. Improving Social Determinants of Health: Effectiveness of a Web-Based Intervention. *Am J Prev Med.* Dec 2015;49(6):822-831.

**5.** Hatef E, Weiner JP, Kharrazi H. A public health perspective on using electronic health records to address social determinants of health: The potential for a national system of local community health records in the United States. *Int J Med Inform.* Apr 2019;124:86-89.

**6.** Henwood BF, Cabassa LJ, Craig CM, Padgett DK. Permanent supportive housing: addressing homelessness and health disparities? *Am J Public Health.* Dec 2013;103 Suppl 2(Suppl 2):S188-192.

**7.** Yaun JA, Rogers LW, Marshall A, McCullers JA, Madubuonwu S. Whole Child Well-Child Visits: Implementing ACEs and SDOH Screenings in Primary Care. *Clin Pediatr (Phila).* Sep 2022;61(8):542-550.

**8.** Stewart de Ramirez S, Shallat J, McClure K, Foulger R, Barenblat L. Screening for Social Determinants of Health: Active and Passive Information Retrieval Methods. *Popul Health Manag.* Dec 2022;25(6):781-788.

**9.** Roebuck E, Urquieta de Hernandez B, Wheeler M, et al. Lessons Learned: Social Determinants of Health Screening Pilot in 2 Urology Clinics. *Urol Pract.* Jan 2022;9(1):87-93.

**10.** Mullen LG, Oermann MH, Cockroft MC, Sharpe LM, Davison JA. Screening for the social determinants of health: Referring patients to community-based services. *J Am Assoc Nurse Pract.* Jul 20 2023.

**11.** Harriett LE, Eary RL, Prickett SA, et al. Adaptation of Screening Tools for Social Determinants of Health in Pregnancy: A Pilot Project. *Matern Child Health J.* Sep 2023;27(9):1472-1480.

**12.** Hao SB, Jilcott Pitts SB, Iasiello J, et al. A Mixed-Methods Study to Evaluate the Feasibility and Acceptability of Implementing an Electronic Health Record Social Determinants of Health Screening Instrument into Routine Clinical Oncology Practice. *Ann Surg Oncol.* Nov 2023;30(12):7299-7308.

**13.** Gupta D, Self S, Thomas D, Supra J, Rudisill C. Understanding the Role of a Technology and EMR-based Social Determinants of Health Screening Tool and Community-based Resource Connections in Health Care Resource Utilization. *Med Care.* Jul 1 2023;61(7):423-430.

**14.** Friedman S, Caddle S, Motelow JE, Meyer D, Lane M. Improving Screening for Social Determinants of Health in a Pediatric Resident Clinic: A Quality Improvement Initiative. *Pediatr Qual Saf.* Jul-Aug 2021;6(4):e419.

**15.** Bradywood A, Leming-Lee TS, Watters R, Blackmore C. Implementing screening for social determinants of health using the Core 5 screening tool. *BMJ Open Qual.* Aug 2021;10(3).

**16.** Page-Reeves J, Kaufman W, Bleecker M, et al. Addressing Social Determinants of Health in a Clinic Setting: The WellRx Pilot in Albuquerque, New Mexico. *J Am Board Fam Med.* May-Jun 2016;29(3):414-418.

**17.** Fleegler E, Bottino C, Pikcilingis A, Baker B, Kistler E, Hassan A. Referral System Collaboration Between Public Health and Medical Systems: A Population Health Case Report. *National Academy of Medicine Perspectivesin the Population Health Case Reports.* 05/27 2016;6.

**18.** Sokol RL, Mehdipanah R, Bess K, Mohammed L, Miller AL. When Families Do Not Request Help: Assessing a Social Determinants of Health Screening Tool in Practice. *J Pediatr Health Care.* Sep-Oct 2021;35(5):471-478.
